# Intra‐ and inter‐observer reliability of ultrasound muscle thickness of gluteal and biceps femoris long head in individuals with and without SCI

**DOI:** 10.1111/cpf.70045

**Published:** 2026-01-08

**Authors:** Boas J. Wijker, Sonja de Groot, Britt Denneman, Puck Brouwer, Vasileios Tomaras, Annelaura Haarler, Guido Weide, Lidwine B. Mokkink, Johanna M. van Dongen, Thomas W. J. Janssen

**Affiliations:** ^1^ Department of Human Movement Sciences, Faculty of Behavioural and Movement Sciences Vrije Universiteit Amsterdam Amsterdam the Netherlands; ^2^ Amsterdam Rehabilitation Research Center, Reade Amsterdam the Netherlands; ^3^ Center of Excellence for Rehabilitation Medicine, University Medical Center Utrecht Brain Center University Medical Center Utrecht and De Hoogstraat Rehabilitation Utrecht the Netherlands; ^4^ Department of Epidemiology and Data Science Amsterdam UMC Amsterdam the Netherlands; ^5^ Amsterdam Public Health Research Institute, Methodology Amsterdam University Medical Center Amsterdam the Netherlands; ^6^ Department of Health Sciences, Faculty of Science Vrije Universiteit Amsterdam Amsterdam the Netherlands

**Keywords:** measurement reliability, measurement reproducibility, muscle morphology, rehabilitation imaging, sonography, skeletal muscle assessment

## Abstract

**Purpose:**

This study aimed to evaluate both inter‐ and intra‐observer reliability of ultrasound‐based muscle thickness measurements in able‐bodied (AB) individuals, as well as intra‐observer reliability in individuals with spinal cord injury (SCI).

**Methods:**

Ultrasound measurements of the gluteus maximus, medius, minimus and biceps femoris long head were performed on 31 AB participants and 30 participants with SCI. Each AB participant was scanned on two occasions by three observers, with three repetitions per muscle per occasion. The muscle thickness in participants with SCI was measured using three repetitions during a single test occasion, conducted by one observer. A generalizability (G) study was conducted to assess the reliability of the measurements.

**Results:**

In AB participants, intra‐observer reliability for gluteal muscles ranged from G‐coefficient: 0.57 to 0.89, and for biceps femoris long head from G‐coefficient: 0.60 to 0.76. Inter‐observer reliability in AB participants was G‐coefficient:0.48–0.72 for the gluteal muscles and G‐coefficient: 0.52 for the biceps femoris. In contrast, intra‐observer reliability in participants with SCI was excellent across all muscles (G‐coefficient: 0.95–0.99).

**Conclusion:**

Ultrasound can assess muscle thickness with moderate to good intra‐observer reliability in AB participants, but with only poor to moderate inter‐observer reliability. In contrast, intra‐observer reliability was excellent in participants with SCI. Reliability depends on observer experience and varies across muscles and populations.

## 1 INTRODUCTION

Depending on the level and completeness of the injury, a spinal cord injury (SCI) can result in muscle atrophy of the paralyzed muscles (Pagano et al., 2018), reduced (micro)circulation, disturbed sympathetic function, and an impaired ability to sense and effectively alleviate pressure (Gilsdorf et al., 1991). One of the most prevalent secondary complications of an SCI is a pressure ulcer, which is damage to the skin and/or subcutaneous tissue. Pressure ulcers typically develop due to prolonged mechanical loading during immobile, weight‐bearing postures (Berg & Ed, 1993), most often in areas where soft tissue lies over bony prominences, such as the ischial tuberosities (Bouten, Oomens, et al., 2003; Gefen et al., 2005).

Sustained compression of the buttocks during sitting compromises perfusion (Jan et al., 2013; Li et al., 2006), leading to ischemia and persistent cellular deformation, which can ultimately result in tissue necrosis and breakdown (Bouten et al., 2003). In seated individuals, peak tissue strains—such as deformations—are typically higher in the gluteal muscles than in the more superficial tissues, including skin and subcutaneous fat (Elsner & Gefen, 2008; Linder‐Ganz et al., 2007; Luboz et al., 2014). Consequently, in individuals with SCI, the gluteal muscles are particularly vulnerable to mechanical loading and may even be at greater risk of damage than the skin itself (Sopher et al., 2010). Pressure ulcers in this population significantly impair quality of life (Roussou et al., 2023) and are associated with high morbidity, increased mortality (Redelings et al., 2005), and substantial healthcare costs (Wijker et al., 2025).

Given the heightened risk of pressure ulcer development in individuals with SCI, there is an urgent need for effective prevention and management strategies.

One promising approach for monitoring muscle health and guiding such strategies is ultrasound imaging. Ultrasound is a noninvasive and relatively low‐cost method for measuring different anatomical structures, including muscle, subcutaneous tissue, nerves, tendons, arteries, and veins. In clinical settings, ultrasound allows healthcare professionals to evaluate muscle changes, such as disuse‐induced atrophy, fatty infiltration or intermuscular adipose tissue, changes that primarily result from inactivity rather than aging. Although measuring muscle thickness using ultrasound is reliable for various quadriceps (Agyapong‐Badu et al., 2014; Ruas et al., 2017; Staehli et al., 2010; Strasser et al., 2013; Thomaes et al., 2012) and biceps femoris (BF) muscles (Palmer et al., 2015; Ruas et al., 2017) in able‐bodied (AB) individuals, there is limited evidence regarding its reliability for assessing gluteal muscle thickness (Aboufazeli et al., 2018; Ikezoe et al., 2011), despite their importance in detecting fatty degeneration, predicting falls in the elderly (Kiyoshige & Watanabe, 2015), and preventing complications in neuromuscular diseases.

The few studies (Aboufazeli et al., 2018; Ikezoe et al., 2011) that did assess the intra‐ or inter‐rater reliability of ultrasound for measuring gluteal muscle thickness in AB individuals, only looked at the entire gluteal muscle group and the gluteus maximus (Gmax), gluteus medius (Gmed) and gluteus minimus (Gmin) have not yet been separately assessed. Two studies conducted in AB individuals and patients in intensive care units reported excellent reliability for the Gmax; however, both studies used scanning protocols that differ from the protocol applied in the present study (Barbalho et al., 2019; Baron et al., 2022). Moreover, both lacked detailed information about the method used to assess reliability and only reported intraclass correlation coefficient (ICC) values. One study, for instance, did not specify who performed the scanning and whether the intra‐reliability or inter‐reliability was investigated (Barbalho et al., 2019). The study by Whittaker et al. examined intra‐rater reliability for ultrasound measurements of the Gmed and Gmin in healthy adolescents and found it to be excellent (Whittaker & Emery, 2014).

Reliability studies on individuals with SCI are scarce. Dudley‐Javoroski et al. (2010) investigated the intra‐ and inter‐observer reliability of monitoring muscle thickness of the vastus lateralis and soleus muscles in people with SCI over time. They reported excellent intra‐observer reliability for both muscles, indicating that repeated measurements by the same observer were highly consistent. However, inter‐observer reliability was only moderate, suggesting that measurement consistency between different operators may depend on the muscle being assessed (Dudley‐Javoroski et al., 2010).

Such studies (Agyapong‐Badu et al., 2014; Kiyoshige & Watanabe, 2015; Palmer et al., 2015; Ruas et al., 2017; Staehli et al., 2010; Strasser et al., 2013; Thomaes et al., 2012) are important, because there are concerns regarding the ability of novice observers to obtain interpretable ultrasound images of muscle tissues in individuals with SCI due to the abnormal echotexture in paralyzed muscles. These muscles often exhibit increased echogenicity, obscured fascial planes, adipose deposition, and fibrosis (Dudley‐Javoroski et al., 2010). This emphasizes the need for a detailed, standardized scanning and analysis protocol and for comparing measurement reliability between AB individuals and those with SCI, as variations in muscle texture may affect image interpretation and measurement consistency. Although ultrasound imaging has shown promise for monitoring muscle changes, few studies have assessed its reliability for evaluating the gluteal muscles in populations with neuromuscular conditions such as SCI.

Reliability and measurement error between ultrasound measurements can be affected at two levels; during image acquisition (i.e., scanning) and image interpretation (i.e., rating). Variation can occur both within and between observers due to several factors, including experience with the scanning technique (e.g., hand position, applied pressure, and probe rotation) (Costa et al., 2009; Erickson, 1997), adherence to standardized scanning protocols, and proficiency in image analysis (e.g., anatomical knowledge, image quality, and software use). These variations influence not only measurement reliability but also measurement error, both of which are crucial for accurate clinical assessments. Reliability studies provide insight into whether a measurement protocol is sufficiently standardized to ensure dependable measurements and can identify potential sources of systematic differences, such as rater‐related effects, which may need to be addressed to improve measurement consistency. Therefore, ensuring reliable ultrasound imaging, especially in populations with SCI, requires proper training, standardized protocols, and observer experience.

This study developed an ultrasound scanning protocol for the Gmax muscle and applied existing protocols, with minor modification for the Gmed, Gmin, and the long head of the BF, based on current literature. Subsequently, the study assessed reliability of measuring the thickness of these muscles in individuals with and without SCI. The ultimate aim was to refine the ultrasound scanning protocol for future research and to provide evidence‐based recommendations that enhance the precision (i.e., measurement error) of muscle thickness assessments using ultrasound.

The aims of this study were:

1. To investigate the intra‐observer reliability of ultrasound measurements of Gmax, Gmed, Gmin and BF muscle thickness in AB participants, and to explore whether observer experience influences reliability.
2. To investigate the intra‐observer reliability of ultrasound measurements of Gmax, Gmed, Gmin and BF muscle thickness in participants with an SCI.
3. To investigate the inter‐observer reliability of ultrasound measurements of Gmax, Gmed, Gmin and BF muscle thickness in AB participants, and to explore whether observer experience influences reliability.

## 2 MATERIALS AND METHODS

### 2.1 Participants

For this study, both AB participants and participants with SCI were recruited.

Able‐bodied participants were recruited through convenience sampling (Etikan, 2016). For them, inclusion criteria were being 18 years of age or older and having no current musculoskeletal injuries affecting the gluteus muscles or BF long head.

Participants with complete or incomplete SCI were recruited from the SCI PREVOLT study (Wijker et al., 2022). For them, inclusion criteria were being aged 18 years or older, having intact gluteal and hamstring muscles and having experienced at least one pressure ulcer in the last 5 years. Exclusion criteria were similar to the SCI PREVOLT study of Wijker et al., i.e., current pressure ulcers in the gluteal or sacral area, or a flaccid paralysis. The SCI PREVOLT study was approved by the Ethics committee of the VUmc, Amsterdam, the Netherlands (reference number: 2020.539). All participants provided written and informed consent prior to participation.

### 2.2 Observers

The ultrasound scans of the AB participants were performed by three observers (BD, VT and BW). All observers received training on the scanning protocol prior to the measurements. Prior to the study, BD (Master student) had no experience with ultrasound, VT had limited experience, but did not use ultrasound routinely (physiotherapist & researcher), and BW (physiotherapist and PhD candidate) had developed the scanning protocol and gained approximately 6 months of daily ultrasound scanning experience among both AB participants and those with SCI. BW was the sole observer for all measurements conducted on people with SCI due to his involvement in the SCI PREVOLT trial. For the remainder of the article, the observers are referred to by number, corresponding to their level of experience: BW as observer 1, VT as observer 2, and BD as observer 3. Observer 2 and observer 3 were jointly introduced to the scanning protocol by observer 1 during a single instructional session. They then engaged in approximately 2 h of hands‐on practice, during which they applied the protocol on each other and a fellow master's student. During this session, they were encouraged to ask questions when needed.

### 2.3 Sample size calculation

The required sample size for both AB participants and those with SCI was calculated based on a target reliability level of 0.75, resulting in a sample size of *N* = 30. To account for potential dropouts, the final sample size was increased to *N* = 34 (Arifin, 2022).

### 2.4 Design

**Figure 1:**
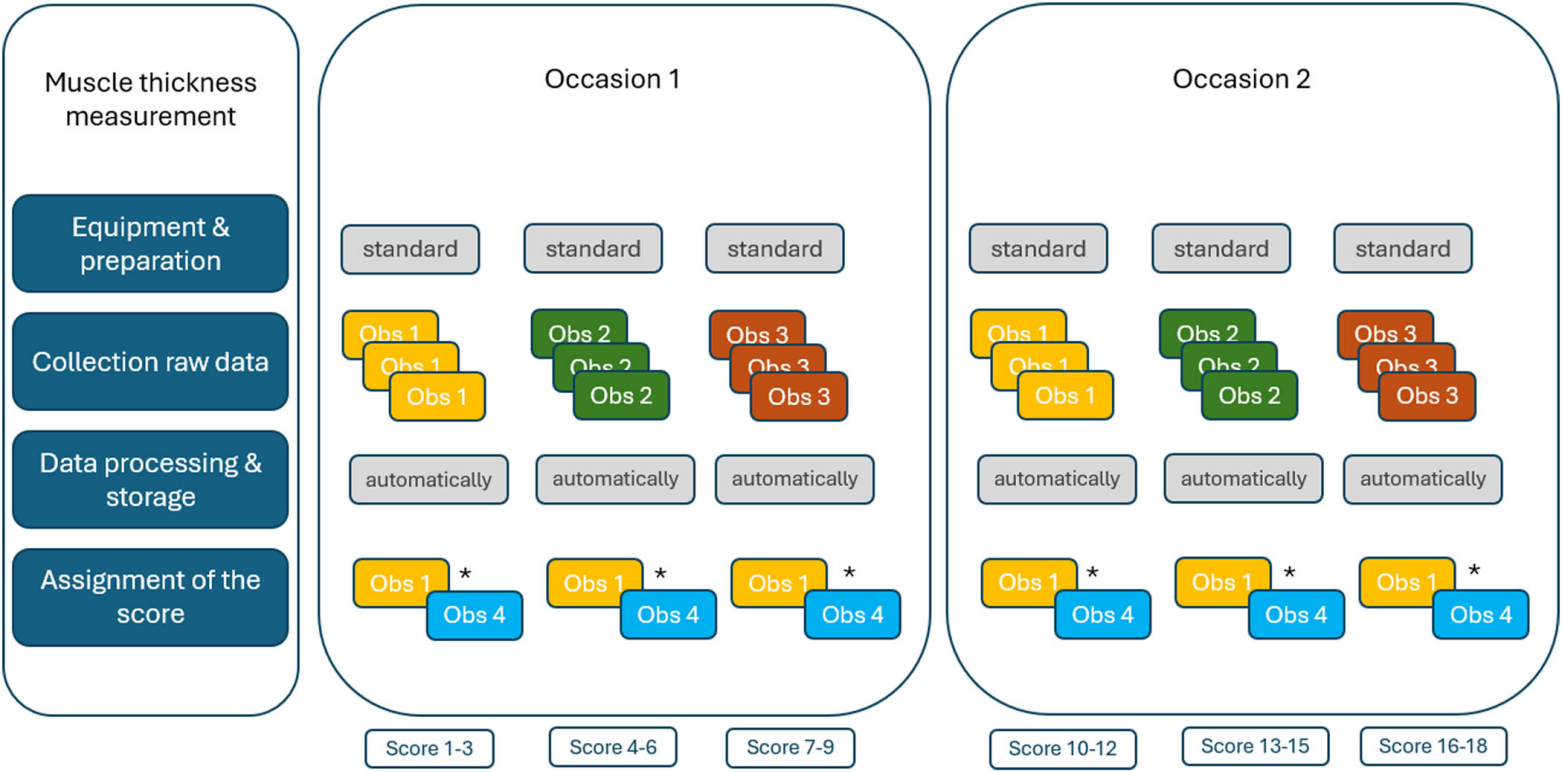
A visual overview of the study design per muscle, for the inter‐observer reliability for the AB participants. Obs 1–4, stands for Observer 1 through 4.

AB participants were scanned by three different observers, with each participant undergoing three repetitions on two separate test occasions at different regions of the body totaling six repetitions for each observer per muscle (see Figure 1). After the first test occasion, AB participants were given 1 h of free time while the observers proceeded to scan the next participant to minimize recall bias. After this hour, the same procedure was repeated (i.e., occasion 2 at Figure 1). For intra‐observer reliability, the same design was used but analyzed for each observer separately (Supporting Information S1: Appendix A).

For each AB participant, 18 muscle thickness measurements per muscle were obtained: 18 for the Gmax, 18 for the Gmed and Gmin (assessed together within a single image), and 18 for the long head of the BF. These measurements were derived from three ultrasound images per muscle, acquired by each observer on two separate test occasions (3 images × 3 observers × 2 occasions). For each muscle, Obs 1 assigned the initial score. Obs 4 (AH), a physiotherapist with approximately 6 months of experience in the scanning protocol and muscle interpretation, independently reviewed these scores to ensure consistency. The detailed procedure for handling discrepancies and reaching consensus is described in the *Image Analysis* section.

For intra‐observer reliability in participants with SCI, only one observer (Obs 1, BW) was involved (see Supporting Information S1: Appendix A). For the participants with SCI, only the baseline measurement of the SCI PREVOLT study was included in the present analysis. During this measurement, three repetitions were done per muscle on a single test occasion.

### 2.5 Ultrasound scanning protocol

All participants were instructed to refrain from exercise or electrical stimulation for at least 24 h prior to the measurement. Participants lay in a supine position with their arms and legs relaxed for a minimum of 10 min to allow stabilization of body fluids (Pinto et al., 2014; Rech et al., 2014). Ultrasound images were obtained using a Philips Lumify L12‐4 transducer (4–12 Mhz) connected to a Samsung galaxy 7+ tablet (Model SM‐T870). Three scanning protocols were used to capture ultrasound images of the Gmax, Gmed, Gmin and the BF muscles. All measurements began with capturing a 3‐second clip, while performing a fan‐shaped movement in the longitudinal plane. This ensured that the transducer was aligned parallel to the muscle fibers and perpendicular to the skin surface, minimizing distortion and enabling an accurate measurement of muscle thickness. One still image was then saved in Brightness‐mode (B‐mode) for offline analysis using MicroDicom viewer.

### 2.6 Gluteus maximus muscle measurements

A literature search was conducted to identify a reliable scanning protocol for this study. However, as no standardized protocol was available, the researchers developed one based on the anatomical properties of the Gmax. The study by Hwang et al. (2009) provided detailed descriptions of muscle thickness of the Gmax, which guided the selection of the thickest part of the Gmax as a reference point.

Since probe movement could affect measurement consistency, different approaches were tested: freehand scanning, reference lines, and bony landmark‐based positioning. To validate the reliability of the portable Lumify device, measurements were compared to those obtained with a higher‐end ultrasound system (Hitachi Arietta Prologue with an L55 5–13 MHz transducer), as well as to direct caliper measurements on a cadaver specimen.

**Figure 2:**
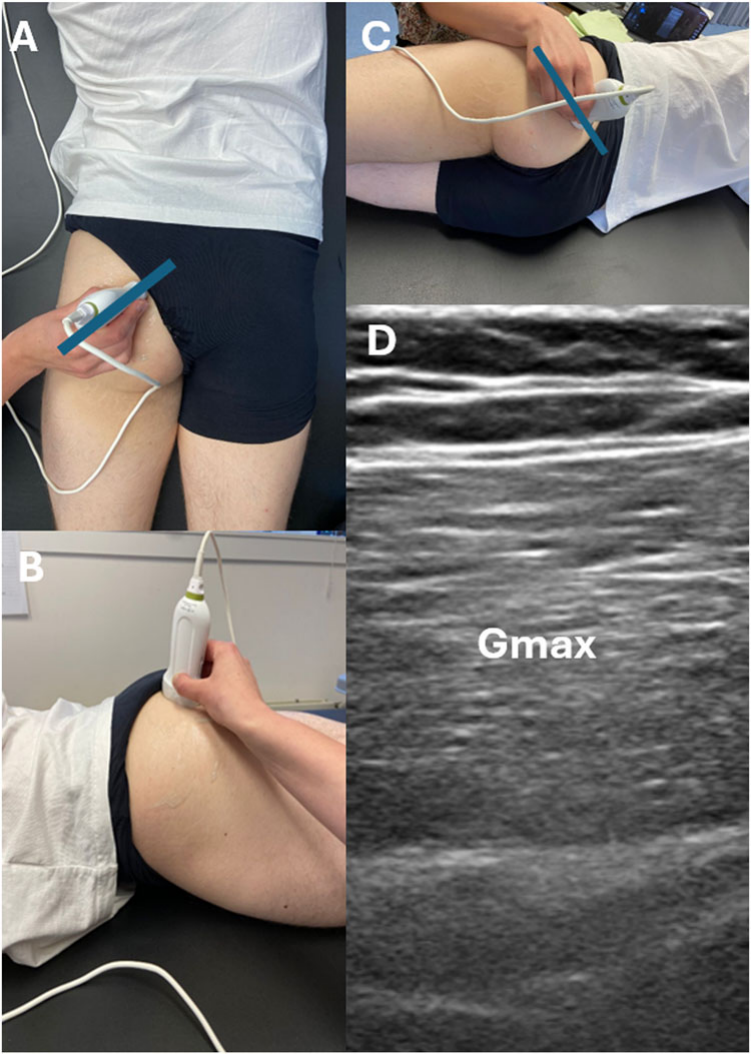
(A) Shows the placement of the probe from the dorsal plane with the reference line in blue, (B) shows the placement of the probe in the transversal plane, (C) shows the placement of the probe after correct positioning the body with the reference line in blue, (D) shows a longitudinal ultrasound image in brightness‐mode of the Gmax (Gmax).

The final scanning protocol was established as follows: Participants were positioned in a lateral position on their left side (Figure 2C the example picture is made on the right side) to ensure consistency in measurements. To determine the Gmax muscle thickness, observers first palpated the greater trochanter and the posterior superior iliac spine. A reference line was then marked between these points using medical tape (see blue line in Figure 2A,C). The ultrasound probe was initially placed on the greater trochanter and moved posteriorly until it reached the thickest part of the Gmax, located in the inner upper quadrant. The probe was then rotated 35–45° to align longitudinally with the muscle fibers.

### 2.7 Gluteus medius and minimus muscle measurements

**Figure 3:**
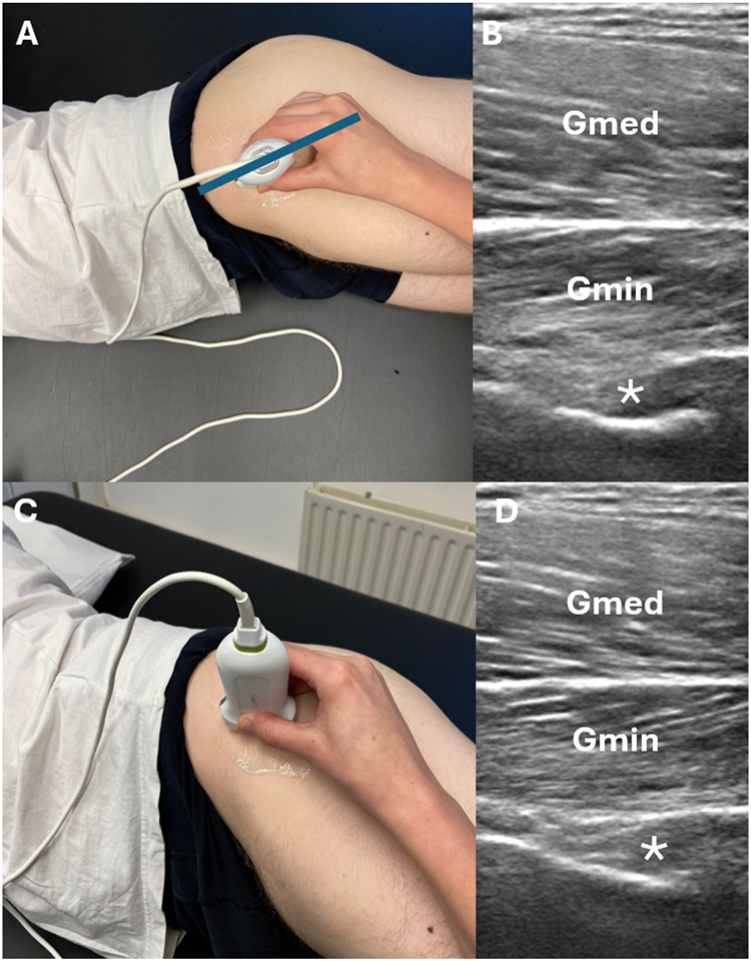
(A) Overview of probe placement, the blue line gives a representation of the angle. (B) Longitudinal ultrasound view of the gluteus medius (Gmed) and gluteus minimus (Gmin), Asterisk * shows the bony landmark. (C) Overview of the probe placement from a frontal‐sagittal view. (D) Shows the longitudinal ultrasound view of the Gmed and Gmin.

This ultrasound scan protocol is based on the method described by Whittaker and Emery (2014). Due to their anatomical proximity, the thicknesses of the Gmed and Gmin muscle can be assessed simultaneously within a single ultrasound image. To obtain this measurement, participants lay on their side and the observer first palpated the greater trochanter. The ultrasound probe was placed longitudinally over the most superficial part of the trochanter major. From this position, the caudal side of the probe (the side facing the trochanter) was rotated 10–15° posteriorly. From there, the probe was then moved a couple of centimetres cranially and medially toward the groin area (Figure 3A,B). At this location, the iliac fossa served as a bony landmark. It appears as a small indentation just above the femoral head and acetabulum on the ultrasound image (Figure 3C,D) (Ikezoe et al., 2011; Perkisas et al., 2021; Whittaker & Emery, 2014). For participants with SCI, the measurement procedure was slightly adapted because participants were not able to lie stably on their side with their legs stretched out. As an alternative, the assessment was performed in a semi‐prone position, approximately 30 degrees from full prone, typically lying on the right side. In this position, the left leg (upper leg) was placed in approximately 30 degrees of hip flexion (anteflexion) with the knee flexed, while the right leg (lower leg) was slightly extended. If extending the right leg triggered spasticity or was limited by contractures, the leg was positioned in greater hip flexion until the participant was lying stably.

### 2.8 Biceps femoris muscle measurements

This ultrasound scan protocol is based on the method described by Balius et al. (2019). For the measurement of the (BF) muscle, participants were instructed to lie in the prone position.

**Figure 4:**
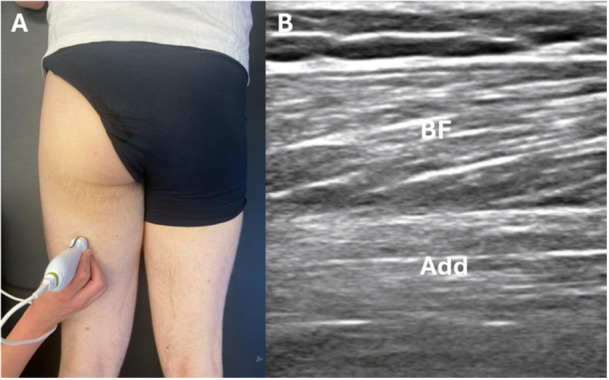
(A) Overview of probe placement, (B) longitudinal ultrasound view of the Biceps femoris (BF: Biceps femoris long head). Add: adductor magnus.

Ultrasound assessment of the long head of the BF began by identifying the ischiatic (sciatic) nerve as a landmark near its origin at the ischial tuberosity, with the transducer initially placed transversely. The transducer was then rotated 90° to obtain a longitudinal view and moved laterally and caudally until it was positioned halfway the muscle belly (Figure 4). If lying in the prone position caused discomfort or was not feasible due to hip contractures, participants with SCI were allowed to assume a semi‐prone position, as described above.

### 2.9 Image analysis

All images were analyzed by one researcher (BW), who also performed the image acquisition. To minimize potential observer bias, an independent researcher (AH, observer 4 in Figure 1) also reviewed all images to ensure consistency in the interpretation of muscle thickness. AH evaluated BW's analyses and assessed whether she agreed with the approach and values used. In cases where discrepancies or disagreements arose, AH explained the reason for her interpretation. These cases were then discussed in a consensus meeting. During this meeting, BW's original measurements were either confirmed as valid, based on a review of the relevant images and ultrasound clips or, if consensus was not reached, the image was re‐analyzed by BW according to the discussion points.

All images were analysed using MicroDicom viewer. Three lines were drawn manually on the ultrasound image. The lines were drawn left, middle, and right, between the superficial and deep aponeuroses, respectively, as illustrated in Supporting Information S1: Appendix B. The measured distances (in centimetres) between the endpoints per line were calculated and the average of the three lines was taken as the muscle thickness. If an aponeurosis was not clearly visible an extra line was drawn where the last muscle fibre direction was visible.

### 2.10 Statistical analysis

Descriptive statistics were calculated for each measured variable and were presented as means with standard deviations (SD), and medians with interquartile ranges (IQR), including the first (Q1) and third quartiles (Q3). These summaries provide insight into data spread and serve as a basis for interpreting measurement error and potential change scores. Intra‐ and inter‐reliability were assessed using a generalization study (G‐study), which estimates reliability and measurement error by partitioning the total variance into components attributable to sources such as participant, repetition, occasion, observer, and their interactions, using a random‐effects Analysis of variance model.

### 2.11 G‐study

A variance component analysis was carried out to obtain the variance components, with a random‐effects design and the method of restricted maximum likelihood. Variance (*σ* ^2^) was calculated for the AB and the SCI participants for the components: participant (*σ* ^2^_p_), repetition (*σ* ^2^_r_), test occasion (*σ* ^2^_ο_), observer (*σ* ^2^_ob_), residual error (*σ* ^2^_res_), and all possible interaction terms. Given the complexity of the study, backward modeling was applied to exclude variables that did not contribute meaningful variance (i.e., those with values close to zero, or those that did not explain variance through interactions), thereby preventing model over‐identification. The highest‐order interaction term participant*repetition*occasion*observer (*σ*²_proob_) was included in the model but should not be confused with the residual variance (*σ*²_res_), which captures random error not explained by any of the modeled effects. To quantify intra and inter‐observer reliability, the G‐coefficient, 95% confidence interval (CI), Standard Error of Measurement (SEM), the Smallest Detectable Change (SDC) and the percentage of SDC% were calculated with the variance components.

The intra‐observer reliability was calculated as follows: (de Vet et al., 2011).

$$G_{\text{coefficients}}{=\sigma }_{p}^{2}{/(\sigma }_{p}^{2}{+\sigma }_{r}^{2}{+\sigma }_{o}^{2}{+\sigma }_{\text{pr}}^{2}{+\sigma }_{\text{po}}^{2}{+\sigma }_{\text{ro}}^{2}{+\sigma }_{\text{res}}^{2}).$$

The inter‐observer reliability, was determined using the same calculation as the intra‐observer reliability but had the addition of the observer variances and their interaction terms to the equation:(de Vet et al., 2011).

$$G_{\text{coefficients}}{=\sigma }_{p}^{2}{/(\sigma }_{p}^{2}{+\sigma }_{r}^{2}{+\sigma }_{o}^{2}{+\sigma }_{\text{ob}}^{2}{+\sigma }_{\text{pr}}^{2}{+\sigma }_{\text{po}}^{2}{+\sigma }_{\text{pob}}^{2}{+\sigma }_{\text{ro}}^{2}{+\sigma }_{\text{rob}}^{2}{+\sigma }_{\text{oob}}^{2}{+\sigma }_{\text{pro}}^{2}{+\sigma }_{\text{prob}}^{2}{+\sigma }_{\text{poob}}^{2}{+\sigma }_{\text{proob}}^{2}{+\sigma }_{\text{res}}^{2}).$$

The corresponding 95% confidence interval (CI) was calculated using the Normalized ICC method specifically the enhanced Wald method to better adjust for potential skewness, small sample bias and makes it possible to use it for the intra‐reliability and the inter‐reliability (Demetrashvili et al., 2016).

The SEM, which provides insight into the precision of individual scores within a patient, was calculated for the measurement error of the intra‐observer study as follows: (de Vet et al., 2011).

$$\text{SEM}=\surd (\sigma_{r}^{2}{+\sigma }_{o}^{2}{+\sigma }_{\text{pr}}^{2}{+\sigma }_{\text{po}}^{2}{+\sigma }_{\text{ro}}^{2}{+\sigma }_{\text{res}}^{2}).$$

And for the inter‐observer measurement error: (de Vet et al., 2011).

$$\text{SEM}=\surd (\sigma_{r}^{2}{+\sigma }_{o}^{2}{+\sigma }_{\text{ob}}^{2}{+\sigma }_{\text{pr}}^{2}{+\sigma }_{\text{po}}^{2}{+\sigma }_{\text{pob}}^{2}{+\sigma }_{\text{ro}}^{2}{+\sigma }_{\text{rob}}^{2}{+\sigma }_{\text{oob}}^{2}{+\sigma }_{\text{pro}}^{2}{+\sigma }_{\text{prob}}^{2}{+\sigma }_{\text{poob}}^{2}{+\sigma }_{\text{proob}}^{2}{+\sigma }_{\text{res}}^{2}).$$

Between models (one‐way, two‐way and three‐way), SEM estimates should be equal, which was used as check for eligibility of the model (Mokkink et al., 2023). The only change in comparison to the inter‐observer reliability formula is the addition of the variance component of the observer and interaction terms.

The SDC, which can be used to monitor clinical changes and serves as a measure of precision around a change score (Weir, 2005), was calculated as follows: (de Vet et al., 2011).

SDC=1.96*√2*SEM

The SDC% was calculated by dividing the SDC by the mean muscle thickness.

All G‐coefficient values were interpreted as follows: poor reliability ≤0.50, moderate reliability 0.5–0.75, good reliability 0.75–0.9, excellent reliability ≥0.9 (Koo & Li, 2016).

In addition, Bland‐Altman plots were generated to visually inspect whether the measurement error was constant across the range of predicted values for each muscle. Instead of using the traditional mean of two measurements on the x‐axis, we used model‐predicted values obtained from the linear mixed model (LMM). The LMM was fitted with muscle thickness as the dependent variable, and observer and participant as random effects, to estimate the predicted values for each measurement. The y‐axis represents the difference between the observed muscle thickness and the corresponding predicted value. This approach was chosen to account for the repeated‐measures design and to retain all repetitions per occasion, without averaging them. All above‐mentioned calculations were conducted in Rstudio (2023.06.1 Build 524© 2009‐2023 Posit Software, PBC).

## 3 RESULTS

The dataset included 61 participants: 31 in the AB group and 30 in the group with SCI. One participant in the AB group was excluded from the analysis for the gluteal muscles but not from the BF due to a high body mass index, which made it impossible to properly interpret the data (Table 1).

**Table 1:** Descriptive statistics.

Categories	AB group (*N* = 31)	SCI group (*N* = 30)
Age: mean years (SD)	35 (18)	56 (11)
Sex; male: n (%)	18 (58%)	25 (83%)
Height (m); mean (SD)	1.79 (0.09)	1.80 (0.07)
Body mass (kg); mean (SD)	74 (11)	85 (18)
BMI (kg/m^2^); mean (SD)	23 (3)	26 (5)
TSI (years); median (IQR)	‐	13 (7 – 23)
Level of SCI: (lumbar/thoracal/cervical)	‐	2/12/16

*Note*: Data are expressed in mean (SD) or n (%).

Abbreviations: BMI, body mass index; IQR, interquartile range; TSI time since injury.

### 3.1 G‐study

#### 3.1.1 Model complexity

During the model construction for both intra‐observer and inter‐observer reliability in the AB group, the results showed no systematic differences (variance) between the repetitions within one occasion Additionally, the model became overidentified when interaction terms involving repetition were included in the four‐way model. This issue was investigated by comparing the mean, SD and the variances of each repetition separately, meaning that repetition one was compared with two, and two with three for each occasion separately. Based on these checks, the model without repetition in the interaction terms was chosen, where only one muscle showed a small variance for repetition (0.00002). Consequently, repetition variance was excluded entirely from all models.

Although the interaction terms including repetitions were excluded from the models, the main effect of repetitions for each occasion was kept in the model to ensure that all sources of variance were accounted for. Using only a single repetition or the mean of all repetitions may influence the measurement error and potentially lead to an overestimation of the G‐coefficient by failing to account for all relevant variance components (de Vet et al., 2011). All other variances and interaction were included in the models.

#### 3.1.2 Intra‐observer reliability

Table 2 provides detailed statistics on muscle thickness [median with interquartile range (IQR)] along with the G‐coefficient (G), SEM, SDC, and the percentage of SDC calculated for all muscles across both the AB and SCI group. This analysis does not account for variance due to repetitions.

**Table 2:** Intra‐observer reliability of ultrasound muscle thickness measurement: two occasions in AB participants and one occasion in participants with SCI.

Muscle	Obs	Mean ± SD (cm)	Median (IQR) (cm)	G	95% CI	SEM	SDC	%SDC
Gmax	1	3.85 ± 0.8	3.67 (3.28–4.24)	0.78	(0.73–0.82)	0.38	1.06	28
Gmax	2	3.85 ± 0.8	3.59 (3.28–4.49)	0.89	(0.84–0.93)	0.27	0.76	20
Gmax	3	3.75 ± 0.72	3.71 (3.25–4.12)	0.60	(0.56–0.64)	0.46	1.28	34
Gmax SCI	1	1.52 ± 0.64	1.41 (1.16–1.80)	0.99	(0.94–1^a^)	0.06	0.17	12
Gmed	1	2.30 ± 0.43	2.3 (2.03–2.58)	0.80	(0.75–0.84)	0.20	0.55	24
Gmed	2	2.32 ± 0.48	2.31 (2.02–2.61)	0.57	(0.53–0.61)	0.32	0.88	38
Gmed	3	2.32 ± 0.66	2.21 (1.85–2.68)	0.89	(0.84–0.93)	0.22	0.62	27
Gmed SCI	1	1.83 ± 0.56	1.74 (1.38–2.10)	0.98	(0.93–1^a^)	0.07	0.20	11
Gmin	1	1.23 ± 0.21	1.25 (1.06–1.36)	0.73	(0.69–0.78)	0.11	0.30	24
Gmin	2	1.19 ± 0.21	1.2 (1.05–1.29)	0.66	(0.62–0.71)	0.13	0.35	29
Gmin	3	1.16 ± 0.22	1.2 (1.02–1.28)	0.76	(0.72–0.81)	0.11	0.31	26
Gmin SCI	1	0.93 ± 0.33	0.92 (0.72–1.19)	0.98	(0.93–1^a^)	0.05	0.14	15
BF	1	2.14 ± 0.54	2.07 (1.7–2.44)	0.76	(0.72–0.81)	0.27	0.74	35
BF	2	2.08 ± 0.40	2.03 (1.85–2.36)	0.60	(0.56–0.64)	0.26	0.72	34
BF	3	2.16 ± 0.40	2.15 (1.9–2.39)	0.63	(0.59–0.67)	0.25	0.68	31
BF SCI	1	1.40 ± 0.31	1.40 (1.23–1.54)	0.95	(0.90–1^a^)	0.07	0.19	14

Abbreviations: 95% CI: confidence interval; G, G‐coefficient; IQR; interquartile range; Obs; observer, mean and SD are given, with median; %SDC = SDC/mean MT.

a Upper confidence intervals were truncated at 1.00 due to the theoretical boundary of the ICC.

In the AB group, the intra‐reliability of the gluteal muscles (Gmax, Gmed and Gmin) and the BF ranged from moderate to good. Comparing the observers, it appears that observer 1, who has the most experience, exhibited the most consistent results across different muscles, achieving an overall moderate to good reliability of 0.73 to 0.80. Observer 2, with some experience, showed moderate to good reliability (0.57–0.89), whereas observer 3, with no prior experience, also displayed moderate to good reliability (0.60–0.89).

In the group with SCI, all muscles demonstrated excellent intra‐reliability. The differences in reliability between the SCI group and the AB group were particularly pronounced for the Gmin and BF. In the AB group, reliability did not exceed 0.76 for any observer, whereas in the SCI group, reliability was substantially higher‐‐0.98 for Gmin and 0.95 for BF.

In the SCI group, participant variance was notably higher, indicating clearer between‐subject differences. In contrast, the AB group showed lower participant variance and relatively more residual and interaction‐related error, particularly for the Gmin and BF. These variance components, presented in Supporting Information S1: Appendix C, suggest that the measurement was more consistent in capturing true inter‐individual differences within the SCI group, which likely contributed to the higher G‐coefficients observed.

#### 3.1.3 Inter‐observer reliability in able‐bodied participants

Table 3 presents the inter‐observer reliability of the muscle thickness measurement for the Gmax, Gmed, Gmin and BF muscles in the AB group.

**Table 3:** Inter‐observer reliability of the muscle thickness measurement in the AB group.

Muscle	Mean ± SD (cm)	Median (IQR) (cm)	G	95% CI	SEM	SDC	%SDC
Gmax	3.82 ± 0.77	3.65 (3.27‐4.3)	0.72	(0.67–0.76)	0.42	1.15	30
Gmed	2.31 ± 0.53	2.28 (1.98‐2.61)	0.48	(0.44–0.52)	0.39	1.07	46
Gmin	1.19 ± 0.22	1.22 (1.04‐1.31)	0.65	(0.61–0.69)	0.13	0.36	30
BF	2.13 ± 0.45	2.07 (1.83‐2.4)	0.52	(0.48–0.56)	0.32	0.88	41

*Note*: Mean and SD are given, with median.

Abbreviations: 95% CI; confidence interval; G, G‐coefficient; IQR, interquartile range; %SDC, SDC/mean MT.

In the AB group, inter‐observer reliability ranged from poor to moderate. Among the muscles assessed, Gmax was the only one to nearly reach good reliability (G‐coefficient: 0.72), whereas the Gmed exhibited the lowest reliability (G‐coefficient: 0.48). Additionally, the %SDC was notably high for most muscles. The %SDC for the Gmax was 30%, indicating an increase in muscle thickness of at least 30% is required to be considered as a real change.

A closer look at the variance components (Supporting Information S1: Appendix C, Table C1) suggests that these differences in reliability are largely driven by low between‐participant variance in certain muscles. For example, Gmin showed minimal participant variance (0.0313) compared to Gmax (0.4396), while still being affected by interaction‐related error (e.g., participant*occasion*observer = 0.0082). This could be interpreted as indicating relatively little variation between participants, which may have made it more difficult to achieve high reliability. The BF displayed similar findings, with modest participant variance and proportionally more variance attributed to observer and interaction effects. These findings underscore that lower reliability values may not solely reflect measurement inconsistency, but also inherent limitations in observable variation for certain muscles in the AB group.

Bland‐Altman plots were generated to visually inspect whether the measurement error was constant across the range of predicted values (see Supporting Information S1: Appendix D). The plots showed no evidence of funnel‐shaped patterns, indicating that measurement error was approximately homoscedastic. Therefore, SEM and SDC were calculated from variance components rather than derived from the Limits of Agreement.

## 4 DISCUSSION

In this generalizability study, we evaluated the intra‐observer reliability of ultrasound assessments for four muscles in both AB participants and participants with SCI, and assessed inter‐observer reliability within the AB group. Intra‐observer reliability in the AB group ranged from moderate to good, with more stable reliability observed in measurements taken by the most experienced observer. In the SCI group, the most experienced observer achieved excellent intra‐observer reliability, suggesting that anatomical and physiological differences in muscle structure between the AB and SCI groups may influence measurement reliability. Inter‐observer reliability in the AB group was notably lower, ranging from poor to moderate. Overall, these findings highlight the importance of observer experience. Muscle‐specific characteristics, such as muscle thickness, depth (deep vs. superficial), fat infiltration, and amount of subcutaneous tissue, appear to contribute to increased variation in intra‐observer ultrasound muscle assessments. The following sections explore these factors in more detail.

The Gmax showed a good intra‐observer reliability for observer one and two, but only moderate reliability for observer three. Observer two was the only one almost reaching excellent reliability. Our results, therefore, do not align with existing literature, as prior studies by Barbalho et al. (2019; Baron et al., 2022) reported excellent reliability using different scanning protocols. However, discrepancies may stem from methodological differences. Both studies used transverse instead of longitudinal scanning, both scanned at 50% of the distance between the greater trochanter and sacrum instead of at the thickest part of the Gmax like our protocol. In addition, Barbalho relied on a single experienced technician without multiple scans, while Baron et al. did not specify the observer's experience. Additionally, both studies lacked reporting on key reliability metrics, such as ICC formulas, 95% CIs, SEM, or SDC, limiting interpretability of their results.

The intra‐observer reliability for the Gmed varied from moderate to good, with only observer three approaching the excellent reliability reported in previous studies (Barbalho et al., 2019; Caputo, 2020). In contrast, Gmin demonstrated more consistent reliability between moderate and good (G‐coefficent between 0.66 and 0.76). Compared with the present study, a more comprehensive protocol was previously implemented, scanning the Gmed transversally and employing a more experienced operator (Aboufazeli et al., 2018). Another study used a standardized protocol with a 20° adduction angle and a younger sample group, which likely enhanced reliability for both muscles (Whittaker & Emery, 2014). Their participants existed of healthy female adolescent soccer players, who likely had lower subcutaneous fat and relatively smaller muscle mass, which may have made anatomical landmarks easier to identify and facilitated more stable measurement. The lower reliability observed in our study, compared to the existing literature, may be attributed to differences in scanning protocols, operator experience, and participant characteristics.

The BF did not exhibit the highest intra‐observer reliability among the muscles measured. This outcome contrasts with previous studies, which reported excellent reliability for the BF muscle thickness (Palmer et al., 2015; Ruas et al., 2017). These previous studies employed experienced sonographers and additional quality control measures. For instance, greater consistency was achieved by averaging two scans, thereby correcting for noise (Palmer et al., 2015). Similarly, high reliability was reported when a standardized protocol was used that involved extensive practice trials and precise landmarking prior to scanning (Freitas et al., 2018). In contrast, our study prioritized protocol reproducibility and included rescanning primarily for image collection, without additional quality checks.

The intra‐observer reliability in the SCI group was excellent across all muscles, likely due to reduced muscle size in the lower extremities, resulting in overall thinner soft tissue layers and improved ultrasound image clarity (Feldman et al., 2009). Although disuse atrophy in individuals with SCI often leads to increased intramuscular fat infiltration and fluid retention (Gorgey et al., 2019), the marked reduction in muscle bulk generally enhances the visibility of bony landmarks and minimizes potential scanning errors. Additionally, a shallower scanning depth and reduced soft tissue mass facilitate image acquisition and improve scanning ease (Feldman et al., 2009). Notably, BMI is not always a reliable predictor of subcutaneous fat thickness, particularly in the anterior thigh region, which can influence ultrasound image quality (Agyapong‐Badu et al., 2014). Variation between muscles persisted due to inherent differences in muscle thickness and composition. Finally, increased familiarity with the protocol, reinforced by a potential learning effect during the study, may have contributed to the high intra‐observer reliability observed.

Inter‐observer reliability showed poor to moderate reliability. The Gmax, Gmin, and BF had moderate reliability, while the Gmed only had poor reliability. Limited literature on gluteal muscle reliability restricts comparisons with our findings. However, one study reported an inter‐observer reliability for the BF of ICC 0.86 (CI 0.55–0.96), representing a combined reliability of both image acquisition and digitizing (Freitas et al., 2018). Unfortunately, we are not able to compare our findings with those of the referenced study, as our analysis focused solely on observer‐specific reliability rather than a combined reliability value. The poor reliability observed for Gmed may, in part, reflect large measurement error between observers rather than low variation between participants. As shown in the variance components (Supporting Information S1: Appendix C, Table A1), the participant variance (0.1378) was roughly equal to the sum of all error components, especially the participant‐by‐observer interaction (0.0865), which suggests inconsistent interpretation between raters. These findings suggest that the poor reliability for Gmed may be more related to observer inconsistency than fundamental measurement issues, highlighting the potential value of improved training.

Comparing intra‐ and inter‐observer reliability in the AB group revealed that inter‐observer reliability was generally lower, with an increasing SEM and SDC accordingly.

This underscores the importance of consistent observer involvement or protocol standardization to ensure measurement accuracy. The Gmax maintained moderate inter‐observer reliability, even exceeding observer three's intra‐observer reliability, suggesting that its scanning protocol may be the most standardized and reproducible across observers. The Gmin and BF long head muscles also showed moderate inter‐observer reliability, though both exhibited declines compared to intra‐observer values. Notably, the Gmin exhibited the lowest SEM and SDC values, as well as the smallest differences between intra‐ and inter‐observer reliability. This is likely due to its proximity to bone, smallest muscle thickness, and possible the scanning location. The Gmed had the greatest difference, dropping from good or moderate intra‐observer reliability (depending on the observer) to poor inter‐observer reliability. Overall, these differences between intra‐ and inter‐observer reliability underscore the differences in reliability between observers and highlight where protocols may be most robust or vulnerable.

Differences in age, sex, and muscle composition may have influenced the reliability of muscle thickness measurements. The reliability was higher in the SCI group than in the AB group. This was unexpected, as we assumed that SCI‐related factors, such as fat infiltration, muscle atrophy, and fibrosis would reduce image clarity. However, the reduced muscle mass in this group may have made it easier to locate bony landmarks, and for the ultrasound probe to capture clear boundaries, compared to the bulkier muscles of AB participants. Additionally, sex‐related differences likely played a role. For example, women often have higher levels of subcutaneous fat, which can obscure muscle boundaries and reduce image clarity. This was particularly visible in the gluteal area and may have contributed to the lower reliability observed in the AB group, which included a higher proportion of female participants. These findings suggest that both biological factors (e.g., muscle size, fat infiltration) and demographic variables (e.g., age and sex) interact in complex ways to influence measurement reliability. A better understanding of these interactions is essential for refining future measurement protocols and improving reliability across diverse populations.

### 4.1 Strength and limitations

In this study, each scan initiated from the same anatomical reference point, following the full scanning protocol for every individual repetition. This approach enhanced reproducibility and represented a notable improvement in the protocol for imaging the Gmax. However, repeated repositioning of the probe may have influenced inter‐observer reliability. Repeating the full scan protocol for each repetition could have introduced more variation than completing the scan protocol once and collecting multiple images without replacing the probe, which could have affected inter‐observer reliability. Another limitation of this study is the apparent learning curve among observers, particularly in the early phase of data collection. Greater variation in measurements was observed during image analysis, particularly among the first 14 participants in the AB group. This likely reflects increasing familiarity with the scanning protocol and anatomical structures over time. Specifically, during image analysis, two main issues were identified that may have influenced both intra‐ and inter‐observer reliability. First, images were interpreted under the assumption that the correct muscle was visible. When the wrong muscle was identified, for example, the semitendinosus was sometimes measured instead of the BF, likely due to anatomical knowledge gaps, this affected reliability. This occurred despite the BF having a distinctive pennation angle and tendon structure. Second, variation in probe and hip angles influenced measurement accuracy in Gmed and Gmin muscle thickness. A more anterior probe placement enhanced the visibility of the Gmin relative to the Gmed, whereas a more posterior placement often captured overlapping tissue from the Gmax, which made it challenging to accurately identify the Gmed. These challenges highlight the importance of hands‐on scanning experience and anatomical understanding for achieving consistent and reliable measurements. Another limitation is the potential observer bias introduced by the same researcher (BW) performing both image acquisition and the muscle thickness measurement. To reduce the risk of observer bias, all analyses were also reviewed by an independent researcher (AH) and any differences in interpretation were discussed until consensus was reached. This may not fully eliminate potential bias, but in the absence of an independent experienced ultrasonographer this setup ensured consistency and represented a realistic approach. Finally, due to ethical and practical considerations, a second measurement session for the SCI group was not conducted. As participants had already completed 2 h of physical testing, additional measurements were avoided to reduce patient burden. This limits the assessment of test–retest reliability in this subgroup.

### 4.2 Recommendations

Based on our findings and existing literature, several recommendations can be made to improve the reliability of ultrasound measurements in both research and clinical practice. Anatomical knowledge, a standardized scanning protocol, familiarity with that specific protocol, and general scanning experience all contribute to more consistent and reliable measurements. In our study, the most experienced observer showed the most consistent reliability across muscles. The literature states that repeated practice reduces measurement error and improves reliability (Warneke et al., 2025). However, other studies mention that reliable images can also be obtained by non‐professional evaluators after brief training sessions of just 10 to 45 min, even without prior ultrasound experience (Carr et al., 2021; Hadda et al., 2018). Some authors describe ultrasound as a feasible and accessible tool that requires minimal training to be used effectively. These contrasting findings make it challenging to define a clear learning curve or to identify the most efficient approach for achieving reliable measurements. In our case, less experienced observers may have benefited from a more extensive training program. For example, Meza‐Valderrama et al. had an extensive approach to the training of observers including; ultrasound techniques, anatomical localization, structure identification, and measurement accuracy (Meza‐Valderrama et al., 2022). These may serve as useful pointers to include in future training protocols. Finally, we recommend further standardization of scanning protocols, particularly for complex muscle groups and when assessments are conducted by less experienced observers. Suggestions include measuring the exact distance to bony landmarks in order achieve consistent and reproducible probe placement. Capturing images of both the patient's position and, for example, the hip angle can be especially helpful—particularly when multiple observers are involved—to ensure consistent replication of scanning conditions.

We recommend the following:

- Involve one experienced sonographer in data collection when possible.
- Multiple repetitions are not necessary to correct for the measurement error when collecting muscle thickness measurement. But when, multiple scans are used, the first could be seen as a test scan and the second repetitions could be used as interpretation. In trials with long follow‐up and multiple test occasions, the emphasis should be on protocol standardization and not on multiple scans per occasion.
- Including a small reliability analysis in all effect studies involving muscle thickness measurements. Reporting ICCs/G‐coefficients, SEM, and SDC enables readers to interpret the results and evaluate clinically important differences.
- When the research staff exist of PhD students or master students, give them extensive training, including: anatomy knowledge, probe handling, and supervised hands‐on practice, while also exploring the surrounding anatomical structures to be able to recognize the correct muscle and its anatomical structures (e.g., pennation angle, tendon, location, etc).
- In clinical settings, ultrasound should be used as an evaluation method only when the practitioner or healthcare professional has received sufficient training and hands‐on experience, supported by clear scanning protocols and standardized analysis procedures.

## 5 CONCLUSION

In conclusion, this intra‐ and inter‐observer reliability study in participants with and without SCI demonstrated that gluteal and BF muscle thickness can be measured with moderate to good intra‐observer reliability, but only poor to moderate inter‐observer reliability when using ultrasound. To maximize consistency and reliability, particularly in studies involving multiple muscles, it is recommended that the same experienced observer performs all measurements. Among the protocols tested, the newly developed scanning protocol for the Gmax demonstrated the highest reproducibility and standardization for both intra‐ and inter‐observer reliability. Future research employing ultrasound to assess muscle thickness should include a preliminary reliability study, as reporting ICC, SEM, and SDC values enhances the credibility of the results and supports a clearer interpretation of findings.

## CONFLICT OF INTEREST STATEMENT

The authors declare no conflicts of interest.

## Supporting information

Supporting information.

Supporting information.

Supporting information.

Supporting information.

## Data Availability

The data that support the findings of this study are available on request from the corresponding author. The data are not publicly available due to privacy or ethical restrictions.
