# Supplementary material for: Intra‐ and inter‐observer reliability of ultrasound muscle thickness of gluteal and biceps femoris long head in individuals with and without SCI
Source: Clin Physiol Funct Imaging. 2026 Jan 8;46(1):e70045. doi: 10.1111/cpf.70045 (PMC12780933; doi:10.1111/cpf.70045)
Supplement: Supplementary file 1 — Supporting information. [file CPF-46-0-s002.docx]

**Appendix A study designs**


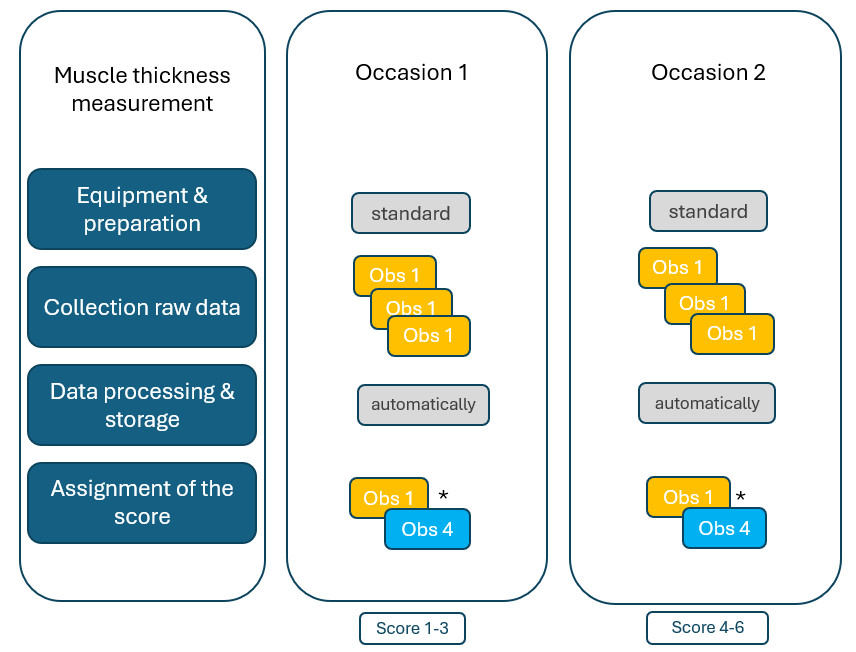


Figure 1b A visible overview of the study design for the intra-observer reliability in the AB participants with observer 1 as an example. Obs 1-4, stands for Observer 1 through 4

**
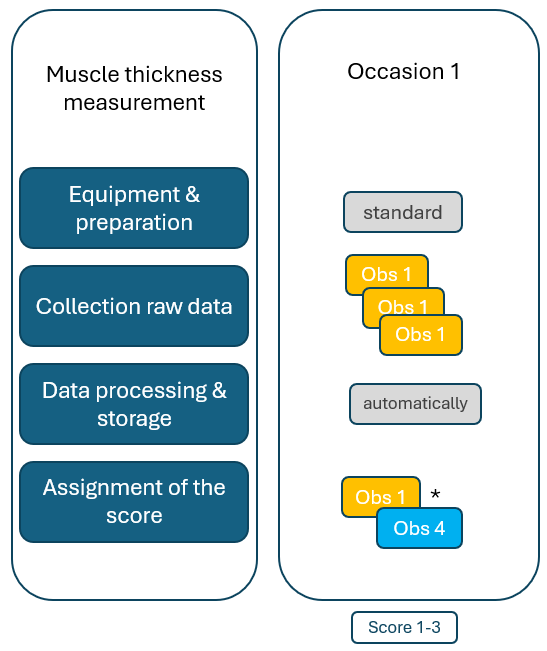
**

Figure 1c visible overview of the study design for the intra-observer reliability in the participants with a SCI. Obs 1-4, stands for Observer 1 through 4
