# Supplementary material for: Intra‐ and inter‐observer reliability of ultrasound muscle thickness of gluteal and biceps femoris long head in individuals with and without SCI
Source: Clin Physiol Funct Imaging. 2026 Jan 8;46(1):e70045. doi: 10.1111/cpf.70045 (PMC12780933; doi:10.1111/cpf.70045)
Supplement: Supplementary file 2 — Supporting information. [file CPF-46-0-s001.docx]

**Appendix B image analysis**


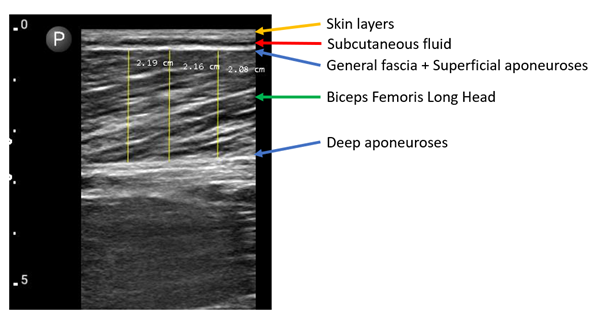


Figure 5. Longitudinal ultrasound view of the BF long head is visible, with a mean muscle thickness of 2.14 cm.
