# Supplementary material for: Intra‐ and inter‐observer reliability of ultrasound muscle thickness of gluteal and biceps femoris long head in individuals with and without SCI
Source: Clin Physiol Funct Imaging. 2026 Jan 8;46(1):e70045. doi: 10.1111/cpf.70045 (PMC12780933; doi:10.1111/cpf.70045)
Supplement: Supplementary file 3 — Supporting information. [file CPF-46-0-s004.docx]

**Appendix C variance components**

The tables below present all variance components from the three-way, two-way, and one-way models. These components were used to calculate the G-coefficient, standard error of measurement (SEM), and smallest detectable change (SDC). The separated variances reflect the sources of measurement error differences between the four muscles, participants, observers, and occasions.

Table C1. Variance components for inter-observer reliability in AB participants.

| **Variance component** | **Gmax** | **Gmed** | **Gmin** | **Ham** |
| --- | --- | --- | --- | --- |
| Participant | 0.4396 | 0.1378 | 0.0313 | 0.1096 |
| Occasion | 0.0004 | 0.0008 | 0.0004 | 0.0000 |
| Observer | 0.0005 | 0.0000 | 0.0008 | 0.0000 |
| Participant*occasion | 0.0246 | 0.0033 | 0.0003 | 0.0074 |
| Participant*observer | 0.0276 | 0.0865 | 0.0028 | 0.0343 |
| Occasion*observer | 0.0000 | 0.0000 | 0.0000 | 0.0020 |
| Participant*occasion*observer | 0.1015 | 0.0391 | 0.0082 | 0.0358 |
| Residual | 0.0189 | 0.0200 | 0.0044 | 0.0206 |

Note: Interaction terms are denoted by “*”, e.g., participant*observer indicates the interaction between participant and observer.

Table C2. Variance components for intra-observer reliability in AB participants and SCI participants with a SCI.

| **Gmax variance component** | **Observer 1** | **Observer 2** | **Observer 3** | **Obs 1 (SCI group)** |
| --- | --- | --- | --- | --- |
| Participant | 0.1317 | 0.0475 | 0.1946 | 0.4204 |
| Occasion | 0.5078 | 0.5777 | 0.3185 |  |
| Participant*occasion | 0.0000 | 0.0021 | 0.0036 |  |
| Residual | 0.0150 | 0.0252 | 0.0163 | 0.0040 |
| **Gmed variance component** | | | | |
| Participant | 0.0181 | 0.0774 | 0.0319 | 0.3106 |
| Occasion | 0.1540 | 0.1347 | 0.3911 |  |
| Participant*occasion | 0.0001 | 0.0023 | 0.0000 |  |
| Residual | 0.0214 | 0.0204 | 0.0183 | 0.0050 |
| **Gmin variance component** | | | | |
| Participant | 0.0071 | 0.0113 | 0.0069 | 0.1104 |
| Occasion | 0.0318 | 0.0316 | 0.0388 |  |
| Participant*occasion | 0.0002 | 0.0011 | 0.0000 |  |
| Residual | 0.0043 | 0.0036 | 0.0053 | 0.0024 |
| **Ham variance component** | | | | |
| Participant | 0.0613 | 0.0462 | 0.0201 | 0.0910 |
| Occasion | 0.2311 | 0.0992 | 0.1035 |  |
| Participant*occasion | 0.0000 | 0.0000 | 0.0090 |  |
| Residual | 0.0102 | 0.0205 | 0.0310 | 0.0048 |

Note: Interaction terms are denoted by “*”, e.g., participant*observer indicates the interaction between participant and observer.
