# Supplementary material for: Intra‐ and inter‐observer reliability of ultrasound muscle thickness of gluteal and biceps femoris long head in individuals with and without SCI
Source: Clin Physiol Funct Imaging. 2026 Jan 8;46(1):e70045. doi: 10.1111/cpf.70045 (PMC12780933; doi:10.1111/cpf.70045)
Supplement: Supplementary file 4 — Supporting information. [file CPF-46-0-s003.docx]

**Appendix D Bland-Altman plots**

Bland-Altman plots (Figures 8–11) were generated for the AB group to visually inspect whether the measurement error was constant across the range of predicted values for each muscle. Predicted values were obtained from a linear mixed model (LMM) that accounted for repeated measurements within participants and across observers, allowing all repetitions per occasion to be retained without averaging. For all four muscles (Gmax, Gmed, Gmin, BF), the differences between observed and predicted values were distributed symmetrically around zero without any clear funnel-shaped patterns. This indicates that the measurement error was approximately homoscedastic across the entire measurement range. Although slightly larger variation was observed for Gmed and BF, these deviations were minor and not systematic. The displayed limits of agreement (LoA) are provided for visual inspection only and were not used to calculate SEM or SDC, which were derived from variance components.


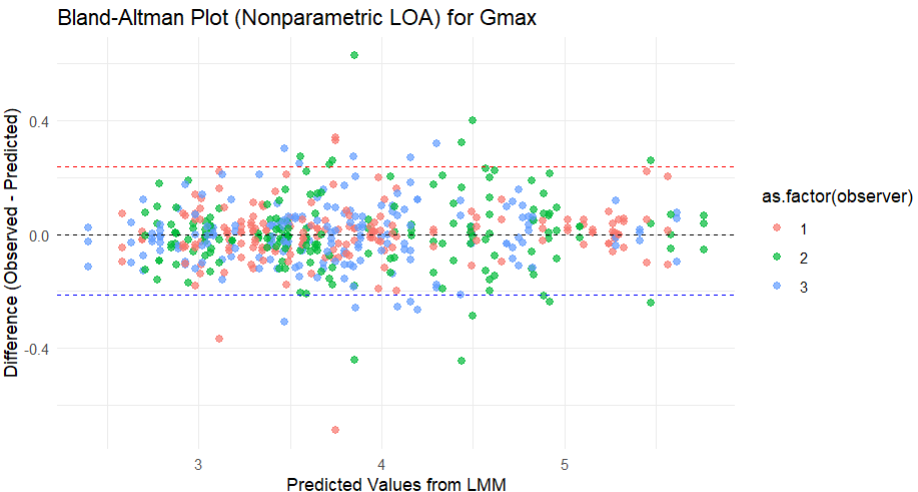


Figure D1. Bland-Altman plot for the Gmax (Gmax), showing the differences between observed and predicted muscle thickness values in AB participants. Predicted values were obtained from a linear mixed model (LMM) that accounted for repeated measurements within participants and across observers. The red and blue dashed lines represent the visual limits of agreement (LoA), while the grey dashed line represents the median difference. These LoA were displayed for visual inspection only and were not used to calculate SEM or SDC.


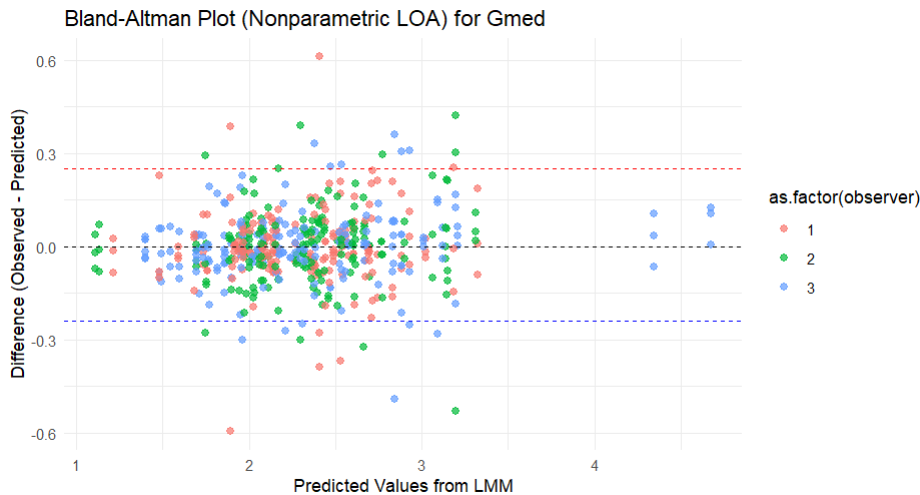


Figure D2. Bland-Altman plot for the gluteus medius (Gmed), showing the differences between observed and predicted muscle thickness values in AB participants. Predicted values were obtained from a linear mixed model (LMM) that accounted for repeated measurements within participants and across observers. The red and blue dashed lines represent the visual limits of agreement (LoA), while the grey dashed line represents the median difference. These LoA were displayed for visual inspection only and were not used to calculate SEM or SDC.


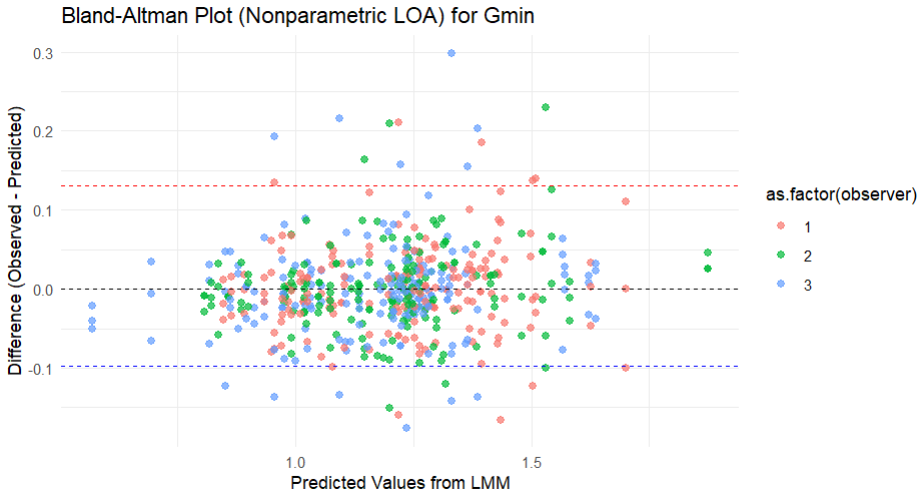


Figure D3. Bland-Altman plot for the gluteus mnimus (Gmin), showing the differences between observed and predicted muscle thickness values in AB participants. Predicted values were obtained from a linear mixed model (LMM) that accounted for repeated measurements within participants and across observers. The red and blue dashed lines represent the visual limits of agreement (LoA), while the grey dashed line represents the median difference. These LoA were displayed for visual inspection only and were not used to calculate SEM or SDC.


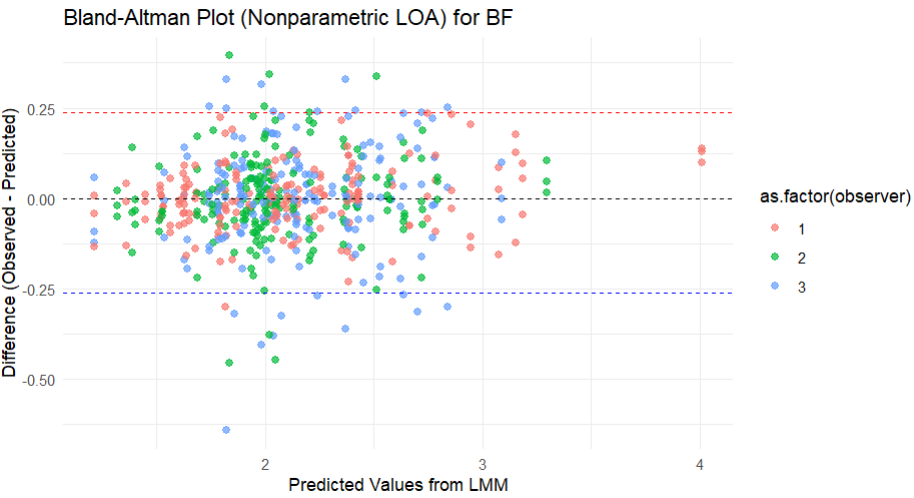


Figure D4. Bland-Altman plot for the biceps femoris (BF), showing the differences between observed and predicted muscle thickness values in AB participants. Predicted values were obtained from a linear mixed model (LMM) that accounted for repeated measurements within participants and across observers. The red and blue dashed lines represent the visual limits of agreement (LoA), while the grey dashed line represents the median difference. These LoA were displayed for visual inspection only and were not used to calculate SEM or SDC.
